# Genome Analysis and Antibiofilm Activity of Phage 590B against Multidrug-Resistant and Extensively Drug-Resistant Uropathogenic *Escherichia coli* Isolates, India

**DOI:** 10.3390/pathogens11121448

**Published:** 2022-11-30

**Authors:** Naveen Chaudhary, Ravi Kumar Maurya, Dharminder Singh, Balvinder Mohan, Neelam Taneja

**Affiliations:** Department of Medical Microbiology, Postgraduate Institute of Medical Education and Research (PGIMER), Chandigarh 160012, India

**Keywords:** phage therapy, urinary tract infections, phage cocktail, multidrug resistance, uropathogenic *Escherichia coli*

## Abstract

Urinary tract infections (UTIs) are among the most common bacterial infections in humans. Uropathogenic *Escherichia coli* (UPEC), which are the most frequent agents causing community as well as hospital-acquired UTIs, have become highly drug-resistant, thus making the treatment of these infections challenging. Recently, the use of bacteriophages (or ‘phages’) against multidrug-resistant (MDR) and extensively drug-resistant (XDR) microorganisms has garnered significant global attention. Bacterial biofilms play a vital role in the pathogenesis of UTIs caused by UPEC. Phages have the potential to disrupt bacterial biofilms using lytic enzymes such as EPS depolymerases and endolysins. We isolated a lytic phage (590B) from community sewage in Chandigarh, which was active against multiple MDR and XDR biofilm-forming UPEC strains. During whole-genome sequencing, the 44.3 kb long genome of phage 590B encoded 75 ORFs, of which 40 were functionally annotated based on homology with similar phage proteins in the database. Comparative analysis of associated phage genomes indicated that phage 590B evolved independently and had a distinct taxonomic position within the genus *Kagunavirus* in the subfamily *Guernseyvirinae* of *Siphoviridae*. The phage disrupted biofilm mass effectively when applied to 24 h old biofilms formed on the Foley silicon catheter and coverslip biofilm models. To study the effect of intact biofilm architecture on phage predation, the biofilms were disrupted. The phage reduced the viable cells by 0.6–1.0 order of magnitude after 24 h of incubation. Regrowth and intact bacterial cells were observed in the phage-treated planktonic culture and biofilms, respectively, which indicated the emergence of phage-resistant bacterial variants. The phage genome encoded an endolysin which might have a role in the disruption and inhibition of bacterial biofilms. Moreover, the genome lacked genes encoding toxins, virulence factors, antibiotic resistance, or lysogeny. Therefore, lytic phage 590B may be a good alternative to antibiotics and can be included in phage cocktails for the treatment of UTIs caused by biofilm-forming MDR and XDR UPEC strains.

## 1. Introduction

Urinary tract infections (UTIs) are the second most frequent bacterial illness and have high morbidity as well as negative impacts on patients’ quality of life and economic status. UTIs are a major concern for both health professionals and patients, as they contribute to nearly 40% of nosocomial infections in hospitals [1]. Uropathogenic *Escherichia coli* (UPEC) is the predominant pathogen causing 40–50% of nosocomial UTIs and 75–85% of community-acquired UTIs [2]. Antibiotics are frequently prescribed by physicians to treat urinary UTIs [3]. UPECs have also evolved as multidrug resistance (MDR) and extended drug resistance (XDR) organisms by becoming resistant to aminoglycosides, third-generation cephalosporins, beta-lactam and beta-lactam inhibitor combinations, ertapenem, and colistin, making treatment difficult. As resistance spreads, there is growing concern that antimicrobial agents will lose efficacy and, in some cases, be rendered ineffective. UPECs have the ability to build biofilms in epithelial cells (intracellular bacterial communities, IBCs) and on urinary catheters, which aids them in resisting host immune responses and treatments [4]. Biofilms are enclosed in extracellular polymeric substances (EPS) that shield the encapsulated microorganisms from the environment [5]. Bacterial biofilms play an important pathogenetic role in causing persistent chronic UTIs, including recurrences and relapses. Biofilm-forming bacteria display some unique properties that differ from those expressed during planktonic growth, including enhanced resistance to antibiotics. Current antibiotics have demonstrated a limited ability to remove biofilms adequately, and phage-based treatments are being presented as viable alternatives for biofilm eradication. Phages exhibit intriguing qualities when it comes to the eradication of biofilms because they produce certain enzymes that enable them to actively infiltrate and disrupt biofilms. Depolymerases and lysins are phage-encoded enzymes that preferentially destroy biofilm EPS matrix components, increasing phage penetration [6].

Moreover, phages may have several advantages over antibiotics. Antibiotics can disrupt gut microbes, causing intestinal homeostasis to be disrupted, whereas phages exhibit lytic activity against pathogenic bacteria while causing minimal harm to the natural flora [6,7]. Phages are capable of multiplying and lysing their bacterial hosts at the site of infection as opposed to antibiotics, which are distributed in all body fluids and tissues based on their intrinsic pharmacokinetic properties rather than being concentrated at the site of infection. Consequently, fewer phage doses are required because of the increase in phage concentration at the site of infection after the initial administration.

Extensive research is being conducted to develop anti-biofilm agents. Recently the FDA approved three phage-based preparations (ListShield^TM^, SalmoFresh^TM^, and EcoShield^TM^) for use in human food applications against biofilm-forming bacterial pathogens *Listeria monocytogens*, *Salmonella enterica*, and *E. coli* [8]. As research advances, the clinical application of bacteriophages should become more standardized and comprehensive. Sequencing the full genome of bacteriophages helps to determine the suitability of the phage for treating bacterial infections.

In this manuscript, we present the genomic characterization and antibiofilm activity of *Escherichia coli* phage 590B, which was isolated from community wastewater around Chandigarh. This phage possesses a strong ability to lyse bacteria in broth culture media and biofilms. Its genome lacks virulence genes, pathogenicity islands, or genes derived from lysogeny or transduction, making it an excellent candidate for phage therapy to treat urinary tract infections caused by biofilm-forming MDR and XDR UPEC isolates.

## 2. Materials and Methods

### 2.1. Sample Collection and Bacterial Strains Used

A total of 65 sewage water samples were collected from four different sewage treatment plants around Chandigarh. Samples were transported to the Enteric laboratory and processed on the same day.

The phage 590B was isolated from a municipal sewage treatment plant in Chandigarh (30°42′43.812″ N 76°48′12.96″ E). The host bacterial strain, UPEC590, was obtained from a clinical case of UTI. The host range of 590B was measured against a library of 50 MDR and XDR UPEC strains using a spot test assay, and the efficiency of plating (EOP) was measured as the ratio of the number of plaques produced with test bacteria and the number of plaques produced with host bacteria. MDR was defined as non-susceptibility to at least one agent in three or more antimicrobials categories of the following classes of antibiotics: third-generation cephalosporins, fluoroquinolones, aminoglycosides, beta-lactamase inhibitor combination, and polymyxins [9]. XDR was defined as non-susceptibility to at least one agent in all but two or fewer above-mentioned antimicrobial categories. All bacterial strains were identified as *E. coli* by employing conventional biochemical tests, followed by matrix-assisted laser desorption ionization time-of-flight (MALDI-TOF, Bruker Daltonics, Bremen, Germany). Serotyping was performed at the Central Research Institute (CRI), Kasauli, India. Virulence genes of phage-sensitive bacterial isolates were conducted using PCR with previously described primers (Table S1) [10]. *E. coli* MTCC 739 obtained from Chandigarh was used as a biofilm-forming control strain. The anti-biofilm activity of the phage was tested against a biofilm-forming clinical strain, UPEC 590.

### 2.2. Phage Isolation and Purification

The raw sewage water samples were centrifuged at 1200 rpm for 15 min to pellet down the heavy debris or particles and followed by filtration with a 0.45 µm membrane filter. The filtrate was mixed with different bacterial cultures in multiple test tubes. After incubation of 18–20 h, the mixture was centrifuged at 4000 rpm and filtered using a 0.22 µm syringe filter. The lytic activity of filtrate was tested against respective bacterial strains using a spot assay. A clear spot area was picked and transferred to the host bacterial culture for overnight incubation at 37 °C. After incubation, a plaque assay was performed, and isolated plaques were picked and incubated with the host bacterial strain. The previous step was repeated thrice to obtain isolated phage plaques. All plates with no bacterial growth in the double-layer plaque assay were scrapped and mixed in sterile SM buffer [11]. The mixture was centrifuged, filtered, and incubated with the addition of 10% of polyethylene glycol 8000. After 24 h, the resultant phage preparation was centrifuged at 55,000 rpm for 2 h using an ultracentrifuge (Beckman Coulter Life Sciences, Brea, CA, USA). Final phage purification was carried out using the CsCl gradient method, followed by dialysis using a dialysis membrane of *MWCO* 12000 (110, HiMedia Laboratories, Mumbai, India). The plaque size was measured and expressed in millimeters.

### 2.3. Phage Morphology

The morphology of the phage was observed using a transmission electron microscope Tecnai G20 (accelerating voltage of 80 Kv) at the All India Institute of Medical Sciences, New Delhi. A drop (approximately 10 µL) of concentrated phage preparation (10^8^ PFU/mL) was applied to a carbon-coated copper grid for 5 min and stained with 4% uranyl acetate (pH 4.0) [12].

### 2.4. One-Step Growth Curve and Adsorption Assay

To evaluate the latent time and burst size of 590B, a one-step growth assay was performed. The host strain was grown to an OD_600_ of approximately 0.5 in 2 mL of trypticase soy broth (TSB) at 37 °C, and the bacterial suspension was diluted further to 10^8^ CFU/mL. The bacterial suspension was mixed with phage suspension at MOI = 0.1 and incubated for 5 min. To eliminate unadsorbed phages, the mixture was centrifuged at 8000 rpm for 5 min. The pellet was resuspended in one ml of the broth, and the resulting mixture was poured into 19 mL of fresh TSB with shaking at 37 °C. Aliquots of one ml were collected at a time interval of 5 min and instantly filtered through a 0.22 µm syringe filter. The phage titer of the supernatant was then determined using a double-layer agar technique. The burst size of 590B phage was determined by dividing the final count of free phage particles by the initial amount of infective bacterial cells [13]. The phage adsorption curve was generated based on the method reported by Chen et al., with some modifications [14]. Briefly, bacterial culture was mixed with the phage at an MOI of 0.1, followed by incubation at 37 °C without shaking. The mixtures were collected every 1 min for 15 min and then centrifuged at 8000 rpm for 5 min. The resultant supernatant was filtered through a 0.22 µm syringe filter, and the phage titer was enumerated as explained above.

### 2.5. Phage Stability

For thermal stability, the phage was added to fresh TSB broth at different temperature conditions (−20 °C, 4 °C, 30 °C, 50 °C, and 60 °C), and for pH stability, it was added to TSB broths having different pH values (pH 4, 5, 6, 7, 8, 9, and 10). The phage titer was measured at an interval of 1 h for 2 h [15].

### 2.6. Genomic DNA Extraction and Sequence Analysis

The genomic DNA was extracted using a phage DNA isolation kit (Norgen Bioteck, Thorold, ON, Canada) [16]. Whole-genome sequencing was executed on the Illumina HiSeq X Ten platform using a paired-end library with a 2 × 150 bp read length. De novo assembly was performed using an IVA v1.0.8 assembler with default k-mer sizes [17]. The genes were predicted from the assembled sequence using GeneMarkSv4.28 and GLIMMER v3.02 systems [18]. The predicted genes were annotated with RAST v2 and searched in the UniProt database. BLASTX hit based on the E-value, identity, and score of each gene was filtered out using the online Uniprot database with default settings. The phage genome was scanned for tRNAs with ARAGORN v1.2.36 [19]. The complete phage genome was scanned with VFDB 2019 (Virulence Factor Database) and CARD (Comprehensive Antibiotic Resistance Database)’s Resistance Gene Identifier (RGI) software v5 CRISPR-Cas Finder (online) to determine virulence factors, antibiotic resistance genes, CRISPR, and CRISPR-like systems in the phage genome, respectively [20,21,22].

### 2.7. Phylogenetic Tree and Comparative Genomics

A neighbor-joining phylogenetic tree (with 500 bootstrap replicates) was constructed based on a large terminase subunit sequence of 590B phage and 19 related phages (BLASTp identity of >99%) using MEGA X [23]. Whole-genome comparison of the 590B phage and four related phages (BLASTn identity >93.83% and query coverage ≥ 70 %) was generated using Mauve v2.4., Easyfig2.2.5, and CoreGenes3.5 [24,25,26]. The whole genome of phage 590B and other sequenced genomes of phages (*n* = 3234) from the virus–host DB (RefSeq release 212) were also compared using ViPTree with default settings [27,28].

### 2.8. Anti-Biofilm Activity of the Phage

**Figure 1:**
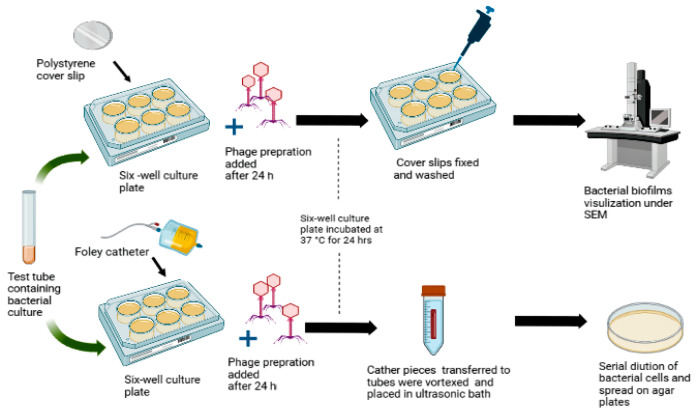
Polystyrene cover slip and Foley catheter biofilm models were used to test the activity of phage 590B against intact biofilms.

The biofilm-forming capacity of the library of 50 UPEC strains was tested using the crystal violet assay [10,29,30]. The quantitative and qualitative antibiofilm activity of the phage 590B was estimated using a Foley silicone catheter and polystyrene coverslips models, respectively. The phages were added after 24 h of bacterial inoculation and incubated for 24 h at 37 °C (Figure 1).

Bacterial biofilms were grown on 20 mm long segments of Foley silicone catheter, which were cut into two halves lengthwise and placed in the six-well culture plates. The phage preparation was added to each treatment group at three MOIs (0.01, 0.1, and 1.0). Further, after 24 h of incubation, the liquid medium of the wells was removed, and catheter segments were washed with PBS buffer. Catheter segments were aseptically transferred to Eppendorf tubes containing 500 µL of 0.8% NaCl, vortexed for one min, and placed into an ultrasonic bath for five min at 35 kHz frequency to remove attached bacterial cells on catheter surfaces. Serial dilutions of bacterial cells were prepared in normal saline and spread onto trypticase soy agar medium.

For bacterial biofilm imaging, biofilms were grown on polystyrene coverslips and treated with phage preparation as described above. After 24 h, the biofilms were fixed with 4% glutaraldehyde for one hour, followed by washing with PBS buffer and dehydration in a series of different concentrations of ethanol, i.e., 50% for 5 min, 70% for 5 min, 90% for 10 min, and 100% for 15 min. Final biofilm visualization was performed using scanning electron microscopy (SEM) [29].

### 2.9. Infection of Disrupted Biofilms with Phage

To understand the impact of intact biofilm architecture on phage predation, 590B was tested in disrupted biofilms. The intact bacterial biofilms were disrupted from the Foley silicon catheter, as described above in Section 2.8. After scraping, biofilms were again infected with phage preparation at MOI 0.01, 0.1, and 1. In the control group, SM buffer was used instead of phage suspension. For viable bacterial count, serial dilution of bacterial cells was prepared in normal saline and spread onto trypticase soy agar medium.

### 2.10. Killing Efficiency of the Phage and Assessment of the Appearance of Phage-Resistant Bacteria

A spectrophotometer-based time-kill assay experiment was performed to evaluate the killing efficiency of the phage. The optical density (OD_600_) was measured at an interval of 2 h for 24 h at three different MOIs, 0.01, 0.1, and 1. The control group contained only bacterial culture grown in TSB media. Both test and control groups were incubated in the same conditions (shaking at 120 rpm under 37 °C). The OD values were measured with a microplate reader (Tecan Group Limited, Mannedorf, Switzerland), and blank values were subtracted from the final values. The phage-resistant bacteria that resulted after 12 and 24 h were diluted to count the viable bacterial cells.

**Figure 2:**
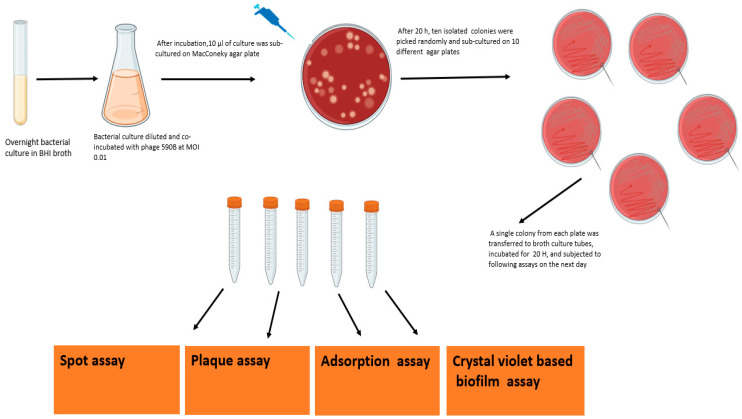
Flow chart showing the procedure to select different phage-resistant mutants and further testing followed by spot assay, plaque assay, adsorption assay, and crystal violet assay.

The phage-resistant variants were isolated, as explained in the flow chart (Figure 2). Briefly, overnight, the bacterial culture was incubated with phage 590B at MOI 0.01. After incubation, 10 isolated colonies were randomly selected and further subjected to spot assay, plaque assay, adsorption assay, and crystal violet-based biofilm assay. Further, to rule out the appearance of a phage tolerance mechanism in bacterial culture, we studied the serial plaque expansion/constriction pattern of the phage 590B against wild UPEC 590 isolate over a period of 20 h as described in a previous study with some modifications [31].

### 2.11. Nucleotide Sequence Accession Number

The genome sequence of phage 590B has been deposited in the GenBank database C with accession no. MW722821.The raw sequencing reads are available at SRA accession no. SRR13926753, BioSample accession no. SAMN17915100, and the BioProject accession no. PRJNA701846.

### 2.12. Statistical Analysis

All statistical tests were performed using Graph pad prism 9.0 software. The Kruskal–Wallis test with the Dunn post hoc multiple comparison test was used for time-kill assay and quantitative biofilm estimation experiments. Statistical analyses for phage stability experiments, one-way ANOVA, and the least significant difference method were applied. *p*-values *** *p* < 0.001, ** *p* < 0.01, and * *p* < 0.05 were considered statistically significant.

## 3. Results

### 3.1. Phage Morphology, One-Step Growth Curve, and Adsorption Kinetics

**Figure 3:**
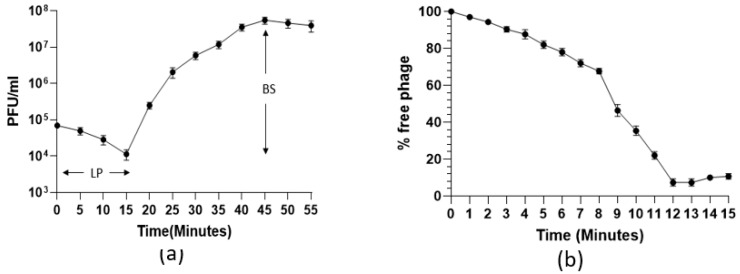
(**a**) One-step growth curve and (**b**) adsorption kinetic curve of phage 590B. The experiments were performed in triplicates, and the error bars indicate the standard deviation. LP: latent period, BS: burst size.

**Figure 4:**
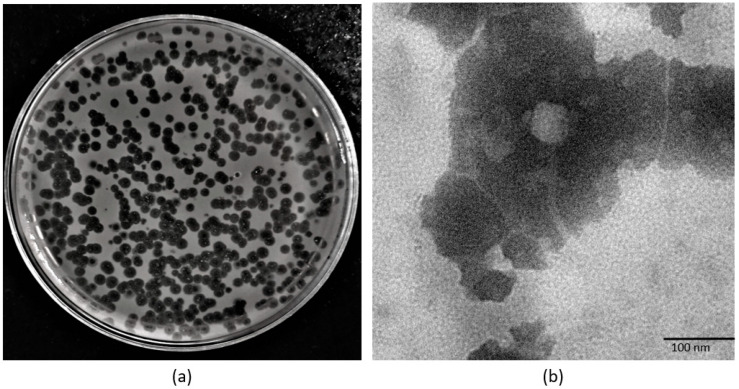
(**a**) Plaque morphology of phage 590B and (**b**) transmission electron micrograph of phage 590B.

The one-step growth curve showed that the 590B phage had a latent period of approximately 15 min and a burst size of approximately 109 phages (Figure 3a). The adsorption kinetic curve demonstrated that the absorption time (95%) of the phage was approximately 12 min (Figure 3b). The phage formed non-turbid plaques (without a halo effect) which were 3–4 mm in diameter (Figure 4a). In the TEM, the phage had an icosahedral head (55 ± 5 nm in diameter) with a long non-contractile tail (190 ± 10 nm) (Figure 4b).

### 3.2. Phage Stability

**Figure 5:**
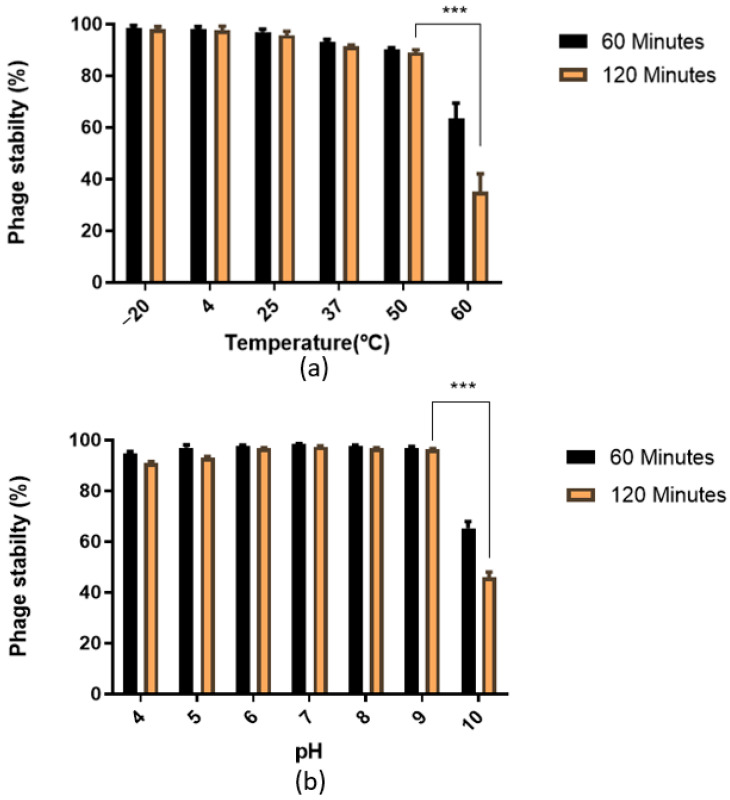
Stability rate of *Escherichia* phage 590B: (**a**) thermal stability and (**b**) pH stability. The phage stability testing experiment was performed in triplicate, and the error bars represent the standard deviation. *** indicates the statistical significance at *p* < 0.001.

The phage 590B was stable at −20 °C, 4 °C, and 25 °C with a >96% stability rate, but the stability significantly (*p* < 0.001) declined sharply to <35% at 60 °C after 2 h (Figure 5a). Similarly, the stability rate of phage was high (>90%) at pH 4 to 9 but significantly (*p* < 0.001) reduced to <47% at pH 10 as compared to the stability rate at pH 9 (Figure 5b).

### 3.3. Genome of Escherichia Phage 590B

**Figure 6:**
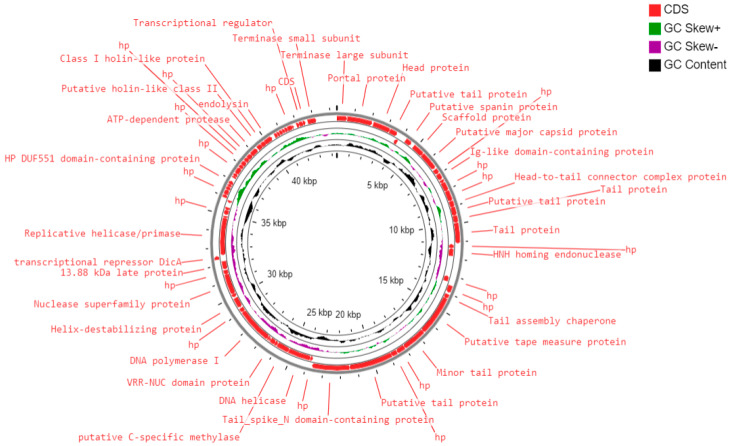
Circular genome map of phage 590B constructed using CG View.

The total genome length of *Escherichia* phage 590B was 44.3 kb, with a GC content of 50.9% (Figure S1). The gene density was calculated to be 1.69. A total of 75 ORFs were discovered, all of which were found on the direct strand (Figure 6). Seventy-three coding sequences were started with the ATG start codon, one with the GTG, and one with the TTG. Table S1 summarizes the positions, lengths, and putative functions of each ORF. Thirty-four ORFs (45.9%) were predicted to encode functional proteins, while forty ORFs (53.3%) were predicted to encode hypothetical proteins. The functional proteins were divided into the following categories.

(i) DNA replication and metabolism proteins: A total of 10 ORFs were predicted to encode for DNA replication and metabolism-related proteins such as HNH homing endonuclease, DNA helicase, putative C-specific methylase, VRR-NUC domain protein, DNA polymerase I, helix-destabilizing protein, nuclease superfamily protein, transcriptional repressor DicA, replicative helicase/primase, helix-turn-helix domain protein, and a transcriptional regulator.
(ii) Structural and packaging proteins: Sixteen ORFs were predicted to encode for structural proteins such as portal protein, head protein, putative tail protein, a scaffold protein, putative major capsid protein, Ig-like domain-containing protein, head-to-tail connector complex protein, putative tail protein, tail protein, tail assembly chaperone, putative tape measure protein, minor tail protein, putative tail protein, tail_spike_N domain-containing protein, and 13.88 kDa late protein. ORF 1 and ORF 75 were predicted to encode for terminase large and small subunits, respectively.
(iii) Host lysis and adhesion proteins: Four ORFs were predicted to encode for putative holins (class-I, II), endolysin, and putative spanin protein. BLASTP analysis of the *Escherichia* phage 590B genome revealed no similarities to genes encoding integrase, recombinase, or excisionase. As a result, phage 590B was classified as a virulent bacteriophage. Furthermore, no result was found against different databases such as VFDB (Virulence Factor Database), Virulence finder 2.0, PAIDB (Pathogenicity Island Database), CARD (Comprehensive Antibiotic Resistance Database), and CRISPRfinder. The genome of phage 590B revealed that it lacks genes encoding for toxins, virulence factors, antibiotic resistance, and CRISPR.

### 3.4. Phylogenetic Analysis and Comparative Genomics

**Figure 7:**
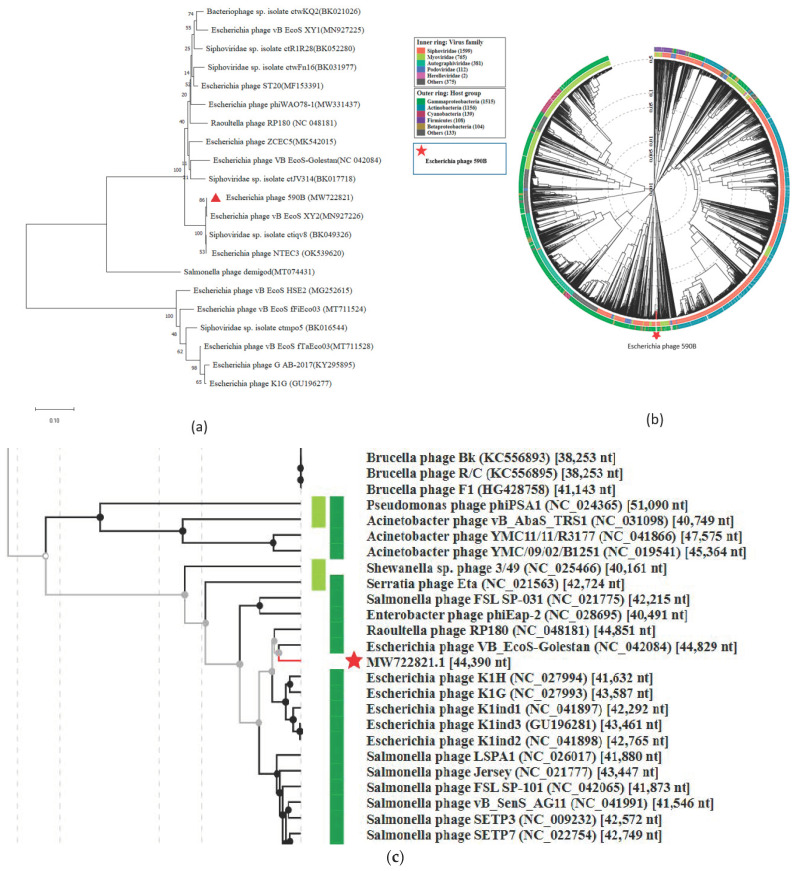
(**a**) The neighbor-joining tree (with 500 bootstrap value) constructed based on the amino acid sequence of the protein of large terminase subunit 590B ( red triangle) and related phages using MEGA-X. (**b**) and (**c**) are circular and rectangular phylogenetic trees generated using ViPTree. Phage 590B (highlighted with a red star) and all sequenced genomes of phages from Virus–host DB (RefSeq release 212) were included in the analysis [27]. The outer and inner rings are colored according to the host group and virus family, respectively.

The phylogenetic tree constructed using a conserved terminase large subunit sequence of 19 closely related published phages revealed 590B grouped with *Escherichia* phage vB_EcoS_XY2 (MN927226) (Figure 7a). To identify the exact taxonomic position of phage 590B, the phylogenetic tree was established using Viptree (Figure 7b,c). Analysis positioned 590B next to the phage VB_EcoS-Golestan (accession no. MG099933) and close to the number of phages with 41–51 kb genomes. Phage 590B was classified as an unclassified *Kagunavirus* in the taxonomical branch *Duplodnaviria* > *Heunggongvirae* > *Uroviricota* > *Caudoviricetes* > *Guernseyvirinae* > *Kagunavirus*.

**Figure 8:**
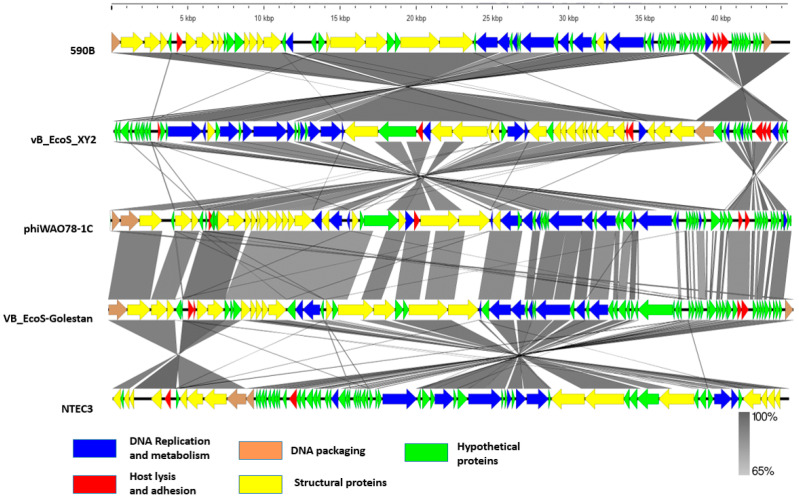
Easyfig image of the genomic comparison of 590B and four related phages. The homologous portions are represented by grey bars, with the color intensity reflecting the degree of sequence similarity.

Genome comparison using MAUVE 2.0 revealed that each phage’s genome contains four local collinear blocks (LCBs), which are areas of sequence homology shared by five phages (Figure S2, Table 1). MAUVE alignment revealed multiple syntenic blocks and appeared to be devoid of internal genome rearrangements. However, some of the blocks revealed genome rearrangements. The black vertical bar indicates a possible orthologous genome section among these five phages. Typically, these are closely linked phage genomes, and the MAUVE alignment reveals large blocks of co-linearization. Easyfig comparisons showed phage genome ORFs oriented and colored differently based on the predicted function of their putative product (Figure 8). For genes encoding a few structural and hypothetical proteins, the degree of nucleotide sequence identity varied. CoreGenes3.5 demonstrated these five phages have 47 protein homologs in total, and *Escherichia* phage 590B had the highest homologs (65.28%) to *Escherichia* phage NTEC3 and the lowest homologs (56.63%) to *Escherichia* phage phiWAO78-1 (Table S3).

### 3.5. Host Range Testing

**Figure 9:**
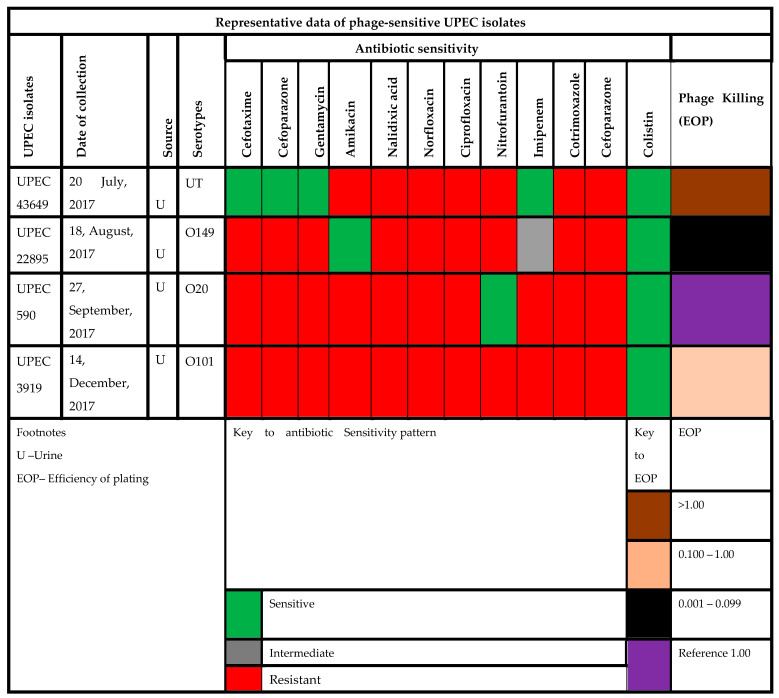
Antibiotic sensitivity and phage killing (EOP) data of phage-sensitive UPEC isolates.

*Escherichia* phage 590B was reported to be active against 8% (4/50) of MDR and XDR UPEC strains in the spot assay (Table S4, Figure S3). Three of the four UPEC strains were related to serotypes O20, O149, and 0101, and one was untypable (Figure 9). The highest EOP (1.2) was found against UPEC 43649, and the lowest EOP (0.088) was against UPEC 22895.

### 3.6. Antibiofilm Activity of Phage 590B

Out of the 50 MDR UPEC strains tested, 24 (48.1%) strains were identified as moderate and 21 strains (42.0%) as strong biofilm-forming isolates. The UPEC 8824 strain was categorized as a non-adherent strain, and the rest of the four strains (8%) were identified as weak biofilm formers (Figure S4). The activity of phage 590B was tested against one of the strong biofilm-forming strains, UPEC 590.

(i) Phage 590B activity against intact and disrupted biofilms formed on the Foley silicon catheter model.

**Figure 10:**
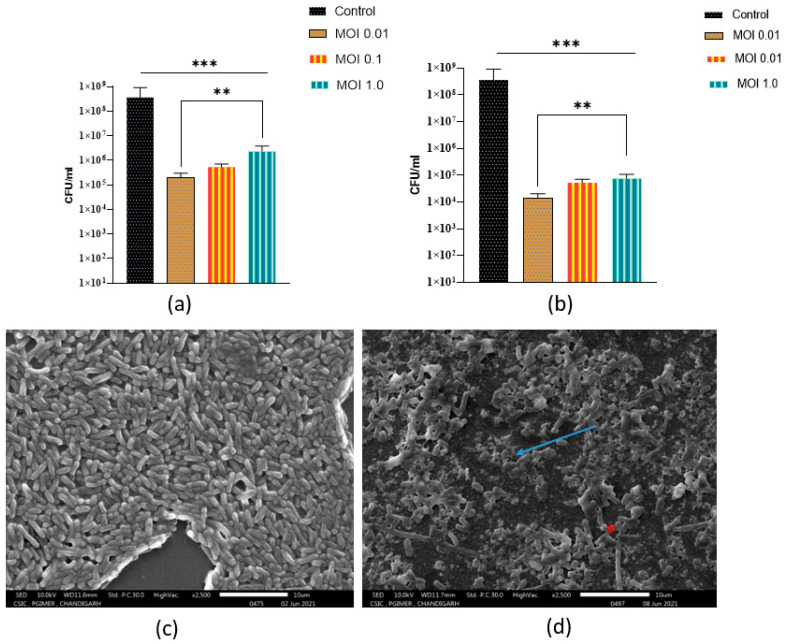
(**a**) The activity of phage 590B against intact 24 h old preformed biofilm formed on Foley silicon catheter and (**b**) disrupted 24 h old preformed biofilm when treated for 24 h (in terms of viable biofilm counts). (**c**) Scanning electron micrograph images (magnification 2500×) of intact 24-old biofilm formed on polystyrene coverslips (control group) and (**d**) treated with phage (10^7^ PFU/mL) for 24 h. Error bars indicate Mean± SD; *p*-values show the significance between control and treated groups. The significant difference indicated by *** *p* < 0.001 and ** *p* < 0.01.

A significant drop (*p* < 0.001) in the biofilm viable counts was observed when 24 old preformed biofilms were treated with phages at three different MOIs as compared to the control group. Again, phage 590B was most effective when the preformed biofilm was treated with phage at MOI 0.01 (Figure 10a).

There was a significant increase in phage efficacy in disrupted biofilms compared to intact biofilms. Phage 590B significantly reduced the number of viable cells in disrupted biofilms after 24 h at three MOIs (approximately 0.6–1.0 orders of magnitude) compared to intact biofilms (Figure 10b).

(ii) Phage activity against intact biofilm formed on polystyrene coverslip

The structural architecture of a typical UPEC biofilm formed on a polystyrene coverslip is depicted in Figure 10c. It shows the bacterial aggregates produce a dense biofilm structure on the coverslip. After phage treatment (10^7^ PFU/mL), reduction of biofilm-forming cells, disruption of the cell surface (blue arrow), and cell integrity were observed, and a large amount of cellular debris was left in the treatment group (Figure 10d). Some intact biofilm-forming bacterial cells were also seen after 24 h of phage treatment (red asterisk).

### 3.7. Appearance of Phage-Resistant Bacteria in Planktonic Culture

**Figure 11:**
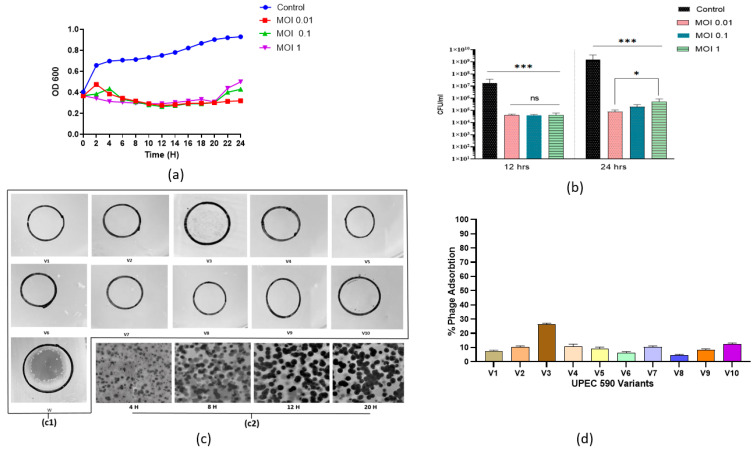
(**a**) Lysis profile (time-kill assay) of MDR UPEC 590 phage and (**b**) the efficiency of formation of phage–resistant bacterial variants during the infection process with the addition of phage 590B at MOI 1.0, MOI 0.1, and MOI 1 after 24 h. (**c**)-(**c1**) Spot assay showing the activity of phage 590B against randomly selected 10 UPEC 590 variants (V1–V10) and 1 wild bacterial UPEC 590 isolate (w). (**c**)-(**c2**) Time-lapse plaque pattern study of phage 590B against wild UPEC 590 isolate. (**d**) Phage adsorption (%) pattern of 10 UPEC590 variants (V1–V10) over 15 min. The above experiments were performed in triplicate. The error bars indicate the standard deviation. The significant difference indicated by * *p* < 0.05 and *** *p* < 0.001. ns. not significant.

When the growth curve was monitored, bacterial density started increasing for the in-itial 4 h at MOI 0.01 and 1 and was significantly reduced by the phage 590B at all three tested MOIs (0.01, 0.1, and 1) used. The phage 590B lytic activity in planktonic culture was evaluated at all three different MOIs (0.01, 0.1, and 1.0) used, and a significant reduction (*p* < 0.05) was detected after 6 h at all MOIs when compared to the control group (Figure 11a). A slight increase in OD values was observed after 20 h at MOIs of 0.1 and 1.0, respec-tively. At MOI 0.01, phage 590B was found to be the most effective where OD values were found to be stable even after 20 h of incubation. When determining the number of viable cells by plating, the bacterial count was approximately similar (*p* > 0.05) at three MOIs (0.01,0.1 and 1.0) after 12 h, while the selection of resistant bacteria was more efficient at high MOI (1.0) than at lower MOIs (0.01 and 0.1) after 24 h of incubation (Figure 11b).

Phage 590B could not form any clear spots or plaques against randomly selected ten bacterial variants though a turbid spot (weak lysis) was observed against one bacterial variant (V3) in the spot assay (Figure 11(c1), Table 2). We did not witness any plaque constriction in our study. However, we observed continuous plaque expansion against wild-type bacterial isolate UPEC 590 tested at four different time points (4, 8, 12, and 20 h). The maximum plaque diameter was reached after 20 h (Figure 11(c2)).

The phage adsorption percentage was significantly reduced (*p* < 0.05) against all of the bacterial variants as compared to the wild bacterial isolate (Figure 11d). Biofilm formation was reduced in the nine variants and increased in variant V1 compared to the wild UPEC 590 strain (Figure S5, Table 2).

## 4. Discussion

Bacterial biofilms are important in the pathogenesis of urinary tract infections (UTIs), not only for catheter-associated infections (CAUTI) but also for persistence, relapses, and acute prostatitis in community-acquired infections. According to the National Institute of Health (NIH), up to 80% of all urological infections are associated with biofilm formation [32]. Urothelium, prostate stones, and foreign bodies that have been implanted can all contain biofilm [33]. Bacteria adhere to the uroepithelium, form a biofilm, and can invade the renal tissue causing pyelonephritis and chronic bacterial prostatitis [34,35]. Biofilms develop on urethral stents and can also cause blockage of catheters. Hence, CAUTI is one of the most common healthcare-associated infections globally [36]. It is challenging to eradicate bacteria present in the biofilms due to the antimicrobial-resistant phenotype that this structure confers. Additionally, persister cells found in biofilms exhibit a slowed metabolism, which raises the levels of AMR [10]. Lytic phages are emerging as promising agents to combat multi-drug-resistant and biofilm-associated infections [37]. Such phages can be combined in cocktails to treat UTIs caused by biofilm-forming drug-resistant UPECs. Previous studies have demonstrated that phages can be utilized to treat acute and chronic inflammatory urologic disorders caused by *E. coli*, *Staphylococcus*, and *Proteus* species, either alone or in conjunction with antibiotics [38]. In order to prepare a cocktail of phages effective against highly drug-resistant MDR and XDR biofilm-forming UPECs, we characterized the phage (590B) which was isolated from a community sewage plant in Chandigarh. We found that this phage was active against UPEC isolates that were resistant to third-generation cephalosporins, fluoroquinolones, aminoglycosides, imipenem, beta-lactamase inhibitor combinations, and polymyxins (Table S4).

We aimed to assess the lytic efficiency of phage 590B against the MDR biofilm-forming UPEC 590 strain obtained from a clinical case of UTI using a Foley silicone catheter and polystyrene cover model. The phage 590B was able to cause disruption of the bacterial surface and biofilm degradation in the polystyrene coverslip model, and the results were in accordance with the quantitative results observed in the Foley silicon catheter model. The efficacy of phage 590B was found to be lower against intact biofilms as compared to disrupted biofilms which might be linked to their complex architecture and the presence of a biofilm matrix [39,40]. The SEM images showed that phage 590B was able to disrupt intact biofilms, but disrupted biofilms still contained small clusters of biofilm-forming communities, indicating a potential role for the matrix in impeding phage efficacy. To investigate this further, an experiment was designed in which biofilm architecture was mechanically disrupted and treated with phage 590B. It was found that cells from disrupted biofilms were more susceptible to the phage using the same infection conditions as those employed against intact biofilms. This may be due to increased interaction between phage and biofilm cells. Further, the slow activity of phages against intact biofilm might also be related to the low metabolic activity of host biofilm-forming bacteria. Bacteria in biofilms grow slowly because they are deprived of nutrients. Phage infection in planktonic bacteria is more efficient than in a biofilm-producing state as more nutrients are available [41]. Phage morphology and biofilm density both influence phage penetration; as biofilm density rises, diffusion becomes more challenging [42].

The biofilm-degrading capability of phages is due to many enzymes capable of degrading biofilm matrix components [43]. Many coliphages, such as T4 and HK620, have enzymes on their tail that aid in bacterial cell wall penetration [44]. Most phages use two key enzymes to remove bacterial biofilms: EPS depolymerase and endolysins [6]. Endolysins tear down the bacterial peptidoglycan structures at the end of the phage growth cycle, allowing phage progeny to be released [45]. ORF 64 of phage 590B was found to have the highest similarity to the endolysin encoding function (identity 98.15%). Endolysins are classified based on cleavage sites, such as lysozymes, N-acetylD-glucosamidases, N-acetylmuramoyl-L-alanine amidases, and L-alanoyl-D-glutamate endopeptidases. Endolysins are typically composed of one of these four N-terminals plus a cell wall-binding domain variant [46]. Exogenously administered lysins can be lytic, and endolysins have been investigated as potential antimicrobials [46]. Recombinant endolysins have recently been studied as potential antibiotic-resistant *Staphylococcus aureus* therapeutics [47]. An important feature of phage 590B was that ORFs 62 and 63 were encoded for class II and class I holins, respectively. Holins are highly charged, hydrophilic proteins that accumulate in the membrane, allowing endolysin to fold to an active state and hydrolyze peptidoglycan cell wall bonds at a specific time [48]. During the lysis process, holins create large holes in the cytoplasmic membrane of bacteria, allowing endolysins to reach and collapse the peptidoglycan structure [45].

Attention has been drawn to phage therapy as an alternative approach to antibiotics, though one of the major obstacles in phage therapy is the regrowth of bacterial subpopulations after phage treatment. We hypothesized that the resurgence of growth in phage-treated bacterial culture was probably either a result of the regrowth of phage-resistant or tolerant subpopulations. The phage tolerance phenomenon is a transient adaptive defense response elicited in the non-infected bacteria upon sensing the infection of their neighbors [31]. Phage tolerance was characterized by a shrinking phase after the initial expansion of the plaques, which was observed at 15 h. We did not witness any plaque constriction in our study. Rather we observed continuous plaque expansion against wild-type bacterial isolate UPEC 590, and the maximum plaque diameter was reached after 20 h. Our study showed that the regrowth of bacteria in the phage-treated culture might be linked to the emergence of phage-resistant mutants, as shown in phage adsorption and spot assay results. Correspondingly, some intact biofilm-forming bacterial cells in the phage-treated group were also witnessed in the SEM results. A previous study revealed that phage selection imposes potential fitness trade-offs between phage resistance and reduced biofilm formation, which was also observed in the majority (9/10) of our selected phage-resistant mutants [49].

The MOI 0.01 was observed to be the most effective in decreasing the bacterial titer in our investigation. Several studies have indicated that phages might be active at different MOIs, and the effective MOIs have ranged from 10^−3^ to 10^5^ [50,51,52]. In our study, the regrowth of bacteria in planktonic culture was more rapid at higher MOI 1.0 and slightly slower at lower MOIs. These findings revealed that the selection of phage-resistant bacteria was more efficient when one bacterial cell was infected by many virions rather than when infection events were rare in the cell population. According to evolutionary theory, mutations arise randomly in populations of any organisms under specific environmental conditions [53]. Bacteria can evolve to possess resistance to phage by mutating receptors on the bactrial cell’s outer membrane, and phage mutants that can use mutated receptors to exploit otherwise resistant bacteria can also evolve resistance [54].

Phage 590B has a high degree of whole-genome sequence similarity (BLASTN identity 93.83–96.68%) to four closely related phages (*Escherichia* phage vBEcoS XY2, *Escherichia* phage VB EcoS-Golestan, *Escherichia* phage phiWAO78-1, and *Escherichia* phage NTEC3), which were isolated from various geographical locations around the world (China, USA, South Korea, India, respectively), implying that these phages have complex evolutionary links. The genome analysis performed using the MAUVE, Easyfig, and CoreGenes3.5 tools revealed that sequence homology was conserved to a greater extent than gene order among the four phages. Phylogenetic analysis (terminase large subunit sequence and ViPTree based) showed that phage 590B belongs to the subfamily *Guernseyvirinae of Siphoviridae* and is far from the other family of phages. The phage 590B was active against several *E. coli* serotypes (O20, O149, O101, and one untypable strain). Phage 590B is an important candidate for inclusion in phage cocktails for potential therapeutic uses due to its ability to elicit lysis of *E. coli* of various serotypes. Additionally, no relationship was discovered between the 590B genome and genes encoding temperate phage markers such as integrase, recombinase, repressor, or excisionase. This indicated the phage had a lytic lifestyle and efficiently produced a high yield of daughter particles (109 PFU/cell). Our phage had a small latent period of 15 min and, combined with a large burst size, has a competitive advantage over other phages as its number can increase in a short time and hence a higher lytic activity is achieved [55,56,57].

Phage 590B remained stable over a wide range of temperatures and pHs. Consequently, it accomplishes essential parameters for survivability and preservation under a wide range of environmental conditions, which will be beneficial for the therapeutic applicability of phage therapy. These results demonstrate the potential of phage 590B to be included in cocktails designed to either prevent or eradicate UPEC biofilms that form on medical devices or intracellular bacterial communities (IBCs) in the urinary tract.

## 5. Conclusions

Phage 590B is a lytic phage from the subfamily *Guernseyvirinae of Siphoviridae* isolated from community sewage water in north India where MDR and XDR UPECs are on the rise. It is effective against multiple MDR and XDR drug-resistant UPEC strains as well as in disrupting bacterial biofilms. Genome characterization revealed that the phage lacks genes of concern, such as genes encoding integrase, recombinase, repressor, and excisionase, as well as virulence factors, antibiotic resistance, and the CRISPR or CRISPR-like system. The phage 590B could be included in cocktails as a safe candidate for phage therapy.

## Figures and Tables

**Table 1:** Genome properties and isolation source of *Escherichia* phage 590B and related four phages.

Phage Name	Taxonomic Genus	Isolation Year	Isolation Source	Genomic Size (bp)	No. of ORFs	Genbank Accession Number
*Escherichia* phage vB_EcoS_XY2	*Kagunavirus*	2020	Sewage water	44138	73	MN927226
*Escherichia* phage VB_EcoS-Golestan	*Kagunavirus*	2020	Sewage water	44829	78	MG099933
*Escherichia* phage phiWAO78-1	*Kagunavirus*	2021	Sewage water	44551	83	MW331437
*Escherichia* phage NTEC3	*Kagunavirus*	2021	Sewage water	44240	74	OK539620
*Escherichia* phage 590B	*Kagunavirus*	2021	Sewage water	44390	52	MW722821

**Table 2:** List of 10 phage-resistant variants according to the phage sensitivity, capacity to form plaques, phage adsorption percentage, and biofilm formation (OD_595_).

UPEC 590B Variants	Phage Sensitivity	Plaque Morphology	% Phage Adsorption	Biofilm Formation (OD_595)_
**V1**	-	NP	7 ± 0.2	1.203 ± 0.04
**V2**	-	NP	10 ± 0.5	0.315 ± 0.07
**V3**	+	NP	25 ± 1	0.503 ± 0.0.01
**V4**	-	NP	12 ± 1	0.405 ± 0.03
**V5**	-	NP	10 ± 0.5	0.301 ± 0.04
**V6**	-	NP	6 ± 0.5	0.410 ± 0.08
**V7**	-	NP	10 ± 0.4	0.308 ± 0.06
**V8**	-	NP	4 ± 0.2	0.405 ± 0.04
**V9**	-	NP	8 ± 0.5	0.400 ± 0.08
**V10**	-	NP	4 ± 0.2	0.402 ± 0.04

Weak lysis (+), no lysis (-), NP: No plaque formation.

## Data Availability

The data presented in this study are available in this article and data used to support the findings of this study are available from the corresponding author upon request.

## Supplementary Materials

The following supporting information can be downloaded at: https://www.mdpi.com/article/10.3390/pathogens11121448/s1, Table S1: Distribution of virulence genes among four host uropathogenic E. coli isolates. Table S2. Functional annotation of phage 590B. Table S3: The relationship of the five phages (Escherichia phage 590B, Escherichia phage vB EcoS XY2, Escherichia phage VB EcoS-Golestan, phiWAO78-1, and NTEC3) at the protein level using CoreGenes3.5. Table S4: Antibiotic sensitivity, the serotyping profile of 50 MDR Uropathogenic E. coli panel strains and host range testing of phage 590B using spot assay. Figure S1: GC content distribution in the genome of phage 590. Figure S2: Genome comparison between phage Escherichia phage 590B and four related phages with Mauve 2.0. The degree of genome sequence similarity between the aligned region is indicated by the height of the similarity profile. The colored blocks are connected by lines depicting the homologous regions among 590B and four related phages. Figure S3: Representative image of spot assay used to test host range of phage 590B. Figure S4: Biofilm formation capacity of 50 MDR, XDR UPEC strains, and MTCC 739 strain in crystal violet assay. Figure S5: Biofilm-forming capacity of ten UPEC590 variants (V1-V10) in crystal violet assay.
