# Supplementary material for: Genome Analysis and Antibiofilm Activity of Phage 590B against Multidrug-Resistant and Extensively Drug-Resistant Uropathogenic Escherichia coli Isolates, India
Source: Pathogens. 2022 Nov 30;11(12):1448. doi: 10.3390/pathogens11121448 (PMC9787291; doi:10.3390/pathogens11121448)
Supplement: Supplementary file 1 [file pathogens-11-01448-s001.zip › pathogens-1891737-supplementary.pdf]

Table S1: Distribution of virulence genes among four host Uropathogenic *E. coli* isolates.

Sr .No.	*E. coli* Strains	UTI-associated *E. coli* genes					Pathotpe
		hlyC	sfa	afaC	papC	fimH	
1	590	-	-	+	+	+	UPEC
2	22895	+	-	+	+	+	UPEC
3	43649	-	-	-	+	+	UPEC
4	3919	+	+	-	+	+	UPEC

Table S2. Functional annotation of phage 590B.

ORF	Start	Stop	Directions	No. of residues	Functional annotation	Identity (%)	Color code
1	2	595	+	197	Terminase large subunit	100% (*Escherichia* phage vB_EcoS_XY2)	
2	608	2089	+	493	Portal protein	100% (*Escherichia* phage vB_EcoS_XY2)	
3	2159	3202	+	347	Head protein	98.3% (*Escherichia* phage vB_EcoS_XY2)	
4	3202	3663	+	153	Putative tail protein	98% (*Escherichia* phage ZCEC5)	
5	3896	3693	-	67	hp	43% (*Salmonella* phage Shemara)	
6	4272	4658	+	128	Putative spanin protein	95.3% (*Escherichia* phage vB_EcoS-Ro145clw*)*	

7	4850	5551	+	233	Scaffold protein	99.6% (*Escherichia* phage vB_EcoS_XY1)	
8	5554	6603	+	349	Putative major capsid protein	99.7% (*Escherichia* phage vB_EcoS_XY2)	
9	6663	6962	+	99	hp	94.9% (*Escherichia* phage ST20)	
10	6975	7328	+	117	Ig-like domain-containing protein	88.9% (*Escherichia* phage ST20)	
11	7365	7544	+	59	hp	100% (*Escherichia* phage VB_EcoS-Golestan)	
12	7548	8060	+	170	hp	100% (*Escherichia* phage VB_EcoS-Golestan)	
13	8063	8677	+	204	hp	96.1% (*Escherichia* phage VB_EcoS-Golestan)	
14	8677	9036	+	119	Head-to-tail connector complex protein	96.6% (*Escherichia* phage vB_EcoS_XY2)	
15	9033	9533	+	166	Putative tail protein	98.8% (*Escherichia* phage vB_EcoS_XY2)	
16	9533	9946	+	137	Tail protein	98.5% (*Raoultella* phage RP180)	

17	9949	11115	+	388	Tail protein	96.9% (*Escherichia* phage vB_EcoS-Ro145clw*)*	
18	11374	11144	-	76	hp	100% (*Escherichia* phage vB_EcoS_HSE2)	
19	11858	11388	-	156	HNH homing endonuclease	98.1% (*Escherichia* phage vB_EcoS-Ro145clw)	
20	13377	13051	-	108	hp	99.1% (*Escherichia* phage vB_EcoS_XY2)	
21	13542	13958	-	138	hp	100% (*Escherichia* phage K1H)	
22	14057	14320	+	87	Tail assembly chaperone	100% (*Escherichia* phage vB_EcoS_XY2)	
23	14313	16625	+	770	Putative tape measure protein	99.4% (*Escherichia* phage vB_EcoS_XY2)	
24	16625	18025	+	466	Minor tail protein	98.9% (*Escherichia* phage vB_EcoS_XY2)	
25	18029	18544	+	171	hp	98.8% (*Escherichia* phage ZCEC5)	
26	18541	18906	+	121	hp	100% (*Escherichia* phage PAB-2017)	
27	18897	21464	+	855	Putative tail protein	98.6% (*Escherichia* phage VB_EcoS-Golestan*)*	

28	21477	23657	+	726	Tail_spike_N domain-containing protein	93.5% (*Escherichia* phage vB_EcoS_XY2)	
29	23823	23686	-	45	hp	100% (*Escherichia* phage P AB-2017)	
30	25244	23820	-	474	DNA helicase	98.1% (*Escherichia* phage *vB_EcoS_fTaEco01)*	
31	26031	25237	-	264	putative C-specific methylase	85.8% (*Escherichia* phage *vB_EcoS_fKuEco01)*	
32	26219	26028	-	63	hp	100% (*Salmonella* phage SS1)	
33	26538	26203	-	110	VRR-NUC domain protein	96.3% *(Escherichia* phage vB_EcoS_XY2)	
34	26751	26620	-	43	hp	100% (*Escherichia* phage VB_EcoS-Golestan)	
35	26623	26525	-	32	hp	100% (*Escherichia phage VB_EcoS*-Golestan)	
36	28924	26741	-	727	DNA polymerase I	97.9% (*Escherichia* phage G AB-2017)	
37	29199	28921	-	92	hp	100% (*Escherichia* phage VB_EcoS-Golestan*)*	

38	29888	29259	-	209	Helix-destabilizing protein	99.5% (*Escherichia* phage vB_EcoS_HSE2)	
39	30164	29961	-	67	hp	98.5% (*Escherichia* phage VB_EcoS-Golestan)	
40	31402	30161	-	413	nuclease superfamily protein	96.6% (*Escherichia* phage G AB-2017)	
41	31671	31399	-	90	hp	91.1% (*Raoultella* phage RP180)	
42	32215	31715	-	166	13.88 kDa late protein	97% (*Escherichia* phage vB_EcoS_XY1)	
43	32340	32555	-	71	Transcriptional repressor DicA	100% (*Escherichia* phage G AB-2017)	
44	34795	32573	-	740	Replicative helicase/primase	99.6% (*Escherichia* phage vB_EcoS_XY2)	
45	34920	34792	-	42	hp	100% (*Escherichia* phage *vB_EcoS_fKuEco01)*	
46	35280	34990	-	96	hp	100 % (*Escherichia* phage vB_EcoS_XY2)	
47	35459	35277	-	60	Helix-turn-helix domain protein	100% (*Escherichia* phage vB_EcoS_fKuEco01)	

48	35884	35732	-	50	hp	26.7% (*Clostridium bornimense*)	
49	35942	36133	-	63	hp	93.7% (*Escherichia* phage vB_EcoS_XY2)	
50	36133	36426	+	97	hp	33% (*Salmonella* phage TS6)	
51	36423	36620	+	65	hp	83.6% (*Raoultella* phage RP180)	
52	36617	36772	+	51	hp	100% (*Escherichia* phage vB_EcoS_fKuEco01*)*	
53	36765	36965	+	66	HP DUF551 domain-containing protein	98.5%(*Escherichia* phage vB_EcoS_fKuEco01*)*	
54	37173	37301	+	42	hp	97.6% (*Escherichia* phage vB_EcoS_fTaEco01)	
55	37291	37479	+	62	hp	88.7% (*Escherichia* phage vB_EcoS_fFiEco02)	
56	37476	37853	+	125	hp	91.4% (*Escherichia* phage P AB-2017)	
57	37855	38058	+	67	hp	91.0% (*Escherichia* phage vB_EcoS_fFiEco02)	
58	38058	38327	+	89	hp	98.9 % (*Escherichia* phage vB_EcoS_XY2)	

59	38327	38578	+	83	hp	74.0 % (*Escherichia* phage vB_EcoS_fKuEco01)	
60	38562	38855	+	97	hp	100 % (*Escherichia* phage vB_EcoS_XY2)	
61	38852	39292	+	146	ATP-dependent protease	99.3% (*Escherichia* phage vB_EcoS_XY2)	
62	39352	39660	+	102	Putative holin-like class II	99% (*Escherichia* phage vB_EcoS-Ro145clw)	
63	39653	39925	+	90	Class I holin-like protein	100% (*Escherichia* phage ZCEC5)	
64	39903	40391	+	162	endolysin	97.5% (*Escherichia* phage vB_EcoS_fFiEco02)	
65	40575	40763	+	62	hp	79% (*Escherichia* phage vB_EcoS_fFiEco02)	
66	40760	40909	+	49	hp	98% (Escherichia phage vB_EcoS_XY2)	
67	40906	41103	+	65	hp	98.5% (*Escherichia* phage vB_EcoS_XY2)	
68	41100	41261	+	53	hp	100%(*Escherichia* phage vB_EcoS_XY2)	
69	41254	41439	+	61	hp	100% (*Escherichia* phage VB_EcoS-Golestan)	

70	41432	41731	+	99	hp	98.0% (*Escherichia* phage vB_EcoS_XY2)	
71	41718	41813	+	31	hp	93.5% (*Escherichia* phage vB_EcoS_XY2)	
72	41988	42158	+	56	hp	100% (*Escherichia* phage vB_EcoS_fKuEco01)	
73	42155	42391	+	78	Transcriptional regulator	100% (*Escherichia* phage vB_EcoS_HSE2)	
74	42388	42543	+	51	hp	100% (*Escherichia* phage vB_EcoS_XY2)	
75	42671	43180	+	169	Terminase small subunit	99.4% (*Escherichia* phage vB_EcoS_XY2)	
						Replication/metabolism	
						Structural and proteins	
						Lysis proteins	
						Packaging proteins	
						Hypothetical proteins (hp)	

Table S3: The relationship of the five phages (*Escherichia* phage 590B, *Escherichia* phage vB EcoS XY2, *Escherichia* phage VB EcoS-Golestan, *Escherichia* phage phiWAO78-1, and *Escherichia* phage NTEC3) at the protein level using CoreGenes3.5.

The number of homologs in each column is :47<br>Total number of genes in genome 1: 75<br>% in common, with respect to genome 1:62.67

Total number of genes in genome 2 :73<br>% in common, with respect to genome 2 :64.38<br>Total number of genes in genome 3:78<br>% in common, with respect to genome 3:60.26<br>Total number of genes in genome 4: 83<br>% in common, with respect to genome 4 :56.63<br>Total number of genes in genome 5:72<br>% in common, with respect to genome 5:65.28				
***Escherichia* phage 590B MW722821**	***Escherichia* phage vB_EcoS_XY2 MN927226**	***Escherichia* phage VB_EcoS-Golestan MG099933**	***Escherichia* phage phiWAO78-1 MW331437**	***Escherichia* phage NTEC3 OK539620**
PI:QVR48582.1 PRODUCT:terminase large subunit	PI:QIG59325.1 PRODUCT:terminase large subunit	PI:ATS93225.1 PRODUCT:putative terminase large subunit	PI:QQV88022.1 PRODUCT:terminase large subunit	PI:UDG73250.1 PRODUCT:terminase large subunit
PI:QVR48583.1 PRODUCT:portal protein	PI:QIG59324.1 PRODUCT:portal protein	PI:ATS93226.1 PRODUCT:putative structural protein	PI:QQV88024.1 PRODUCT:portal protein	PI:UDG73249.1 PRODUCT:62 kDa structural protein
PI:QVR48584.1 PRODUCT:head protein	PI:QIG59323.1 PRODUCT:head protein	PI:ATS93227.1 PRODUCT:hypothetical protein	PI:QQV88027.1 PRODUCT:head morphogenesis protein	PI:UDG73248.1 PRODUCT:head protein
PI:QVR48585.1 PRODUCT:putative tail protein	PI:QIG59322.1 PRODUCT:tail protein	PI:ATS93228.1 PRODUCT:putative tail protein	PI:QQV88026.1 PRODUCT:tail protein	PI:UDG73247.1 PRODUCT:fibritin protein
PI:QVR48586.1 PRODUCT:putative spanin protein	PI:QIG59320.1 PRODUCT:putative spanin	PI:ATS93231.1 PRODUCT:putative spanin	PI:QQV88030.1 PRODUCT:putative spanin	PI:UDG73245.1 PRODUCT:putative spanin protein
PI:QVR48587.1 PRODUCT:scaffold protein	PI:QIG59318.1 PRODUCT:scaffold protein	PI:ATS93233.1 PRODUCT:hypothetical protein	PI:QQV88034.1 PRODUCT:scaffold protein	PI:UDG73244.1 PRODUCT:scaffold protein
PI:QVR48590.1 PRODUCT:Ig-like domain-containing protein	PI:QIG59315.1 PRODUCT:capsid decoration protein	PI:ATS93235.1 PRODUCT:hypothetical protein	PI:QQV88037.1 PRODUCT:capsid decoration protein	PI:UDG73242.1 PRODUCT:neck whiskers protein
PI:QVR48591.1 PRODUCT:hypothetical protein	PI:QIG59314.1 PRODUCT:head-tail joining protein	PI:ATS93236.1 PRODUCT:hypothetical protein	PI:QQV88038.1 PRODUCT:head-to-tail joining protein	PI:UDG73241.1 PRODUCT:hypothetical protein
PI:QVR48592.1 PRODUCT:hypothetical protein	PI:QIG59313.1 PRODUCT:head-to-tail connector complex protein	PI:ATS93237.1 PRODUCT:hypothetical protein	PI:QQV88039.1 PRODUCT:head-to-tail connector complex protein	PI:UDG73240.1 PRODUCT:head-to-tail connector complex protein
PI:QVR48594.1 PRODUCT:head-to-tail connector complex	PI:QIG59311.1 PRODUCT:head-to-tail connector	PI:ATS93239.1 PRODUCT:hypothetical protein	PI:QQV88041.1 PRODUCT:head-to-tail connector complex protein	PI:UDG73311.1 PRODUCT:head-to-tail connector

	complex protein			
PI:QVR48595.1 PRODUCT:putative tail protein	PI:QIG59310.1 PRODUCT:putative tail protein	PI:ATS93240.1 PRODUCT:putative tail protein	PI:QQV88042.1 PRODUCT:tail protein	PI:UDG73310.1 PRODUCT:tail protein
PI:QVR48596.1 PRODUCT:tail protein	PI:QIG59309.1 PRODUCT:tail protein	PI:ATS93241.1 PRODUCT:hypothetical protein	PI:QQV88043.1 PRODUCT:tail protein	PI:UDG73309.1 PRODUCT:tail protein
PI:QVR48597.1 PRODUCT:tail protein	PI:QIG59308.1 PRODUCT:putative tail protein	PI:ATS93242.1 PRODUCT:putative tail protein	PI:QQV88044.1 PRODUCT:tail tube protein	PI:UDG73308.1 PRODUCT:putative tail protein
PI:QVR48598.1 PRODUCT:hypothetical protein	PI:QIG59304.1 PRODUCT:tail assembly chaperone	PI:ATS93247.1 PRODUCT:hypothetical protein	PI:QQV88049.1 PRODUCT:tail assembly chaperone	PI:UDG73303.1 PRODUCT:hypothetical protein
PI:QVR48599.1 PRODUCT:tail assembly chaperone	PI:QIG59303.1 PRODUCT:tail assembly chaperone	PI:ATS93248.1 PRODUCT:hypothetical protein	PI:QQV88050.1 PRODUCT:hypothetical protein	PI:UDG73302.1 PRODUCT:hypothetical protein
PI:QVR48600.1 PRODUCT:putative tape measure protein	PI:QIG59302.1 PRODUCT:putative tape measure protein	PI:ATS93249.1 PRODUCT:putative tape measure protein	PI:QQV88051.1 PRODUCT:putative tail tape measure protein	PI:UDG73301.1 PRODUCT:tail tape measure protein
PI:QVR48601.1 PRODUCT:minor tail protein	PI:QIG59301.1 PRODUCT:minor tail protein	PI:ATS93250.1 PRODUCT:hypothetical protein	PI:QQV88052.1 PRODUCT:minor tail protein	PI:UDG73300.1 PRODUCT:hypothetical protein
PI:QVR48602.1 PRODUCT:hypothetical protein	PI:QIG59300.1 PRODUCT:transcriptional regulator	PI:ATS93251.1 PRODUCT:hypothetical protein	PI:QQV88053.1 PRODUCT:enolase-like protein	PI:UDG73299.1 PRODUCT:hypothetical protein
PI:QVR48603.1 PRODUCT:hypothetical protein	PI:QIG59299.1 PRODUCT:peptidoglycan endopeptidase	PI:ATS93252.1 PRODUCT:hypothetical protein	PI:QQV88054.1 PRODUCT:peptidoglycan endopeptidase	PI:UDG73298.1 PRODUCT:hypothetical protein
PI:QVR48604.1 PRODUCT:putative tail protein	PI:QIG59298.1 PRODUCT:putative tail protein	PI:ATS93253.1 PRODUCT:putative tail protein	PI:QQV88055.1 PRODUCT:putative tail fiber protein	PI:UDG73297.1 PRODUCT:tail protein
PI:QVR48605.1 PRODUCT:tail spike N domain-containing protein	PI:QIG59297.1 PRODUCT:hypothetical protein	PI:ATS93254.1 PRODUCT:tail fibers protein	PI:QQV88057.1 PRODUCT:tail spike protein	PI:UDG73296.1 PRODUCT:tail spike protein
PI:UCR96559.1 PRODUCT:hypothetical protein	PI:QIG59296.1 PRODUCT:exonuclease subunit	PI:ATS93255.1 PRODUCT:exonuclease subunit	PI:QQV88056.1 PRODUCT:exonuclease subunit	PI:UDG73295.1 PRODUCT:hypothetical protein

PI:UCR96560.1 PRODUCT:DNA helicase	PI:QIG59295.1 PRODUCT:helicase	PI:ATS93257.1 PRODUCT:helicase	PI:QQV88059.1 PRODUCT:ATP-dependent helicase	PI:UDG73294.1 PRODUCT:DNA helicase
PI:UCR96562.1 PRODUCT:hypothetical protein	PI:QIG59293.1 PRODUCT:hypothetical protein	PI:ATS93259.1 PRODUCT:hypothetical protein	PI:QQV88062.1 PRODUCT:hypothetical protein	PI:UDG73292.1 PRODUCT:hypothetical protein
PI:UCR96563.1 PRODUCT:VRR-NUC domain protein	PI:QIG59292.1 PRODUCT:VRR-NUC domain protein	PI:ATS93260.1 PRODUCT:VRR-NUC domain protein	PI:QQV88063.1 PRODUCT:endonuclease	PI:UDG73291.1 PRODUCT:endonuclease protein
PI:UCR96564.1 PRODUCT:hypothetical protein	PI:QIG59290.1 PRODUCT:hypothetical protein	PI:ATS93262.1 PRODUCT:hypothetical protein	PI:QQV88067.1 PRODUCT:hypothetical protein	PI:UDG73289.1 PRODUCT:hypothetical protein
PI:UCR96566.1 PRODUCT:DNA polymerase I	PI:QIG59288.1 PRODUCT:DNA polymerase	PI:ATS93263.1 PRODUCT:putative DNA polymerase	PI:QQV88068.1 PRODUCT:thermostable DNA polymerase I	PI:UDG73288.1 PRODUCT:DNA polymerase I
PI:UCR96568.1 PRODUCT:helix-destabilizing protein	PI:QIG59287.1 PRODUCT:helix-destabilizing protein	PI:ATS93265.1 PRODUCT:hypothetical protein	PI:QQV88069.1 PRODUCT:helix-destabilizing protein	PI:UDG73287.1 PRODUCT:hypothetical protein
PI:UCR96570.1 PRODUCT:nuclease superfamily protein	PI:QIG59285.1 PRODUCT:PD-(D/E)XK nuclease superfamily protein	PI:ATS93267.1 PRODUCT:PD-(D/E)XK nuclease superfamily protein	PI:QQV88071.1 PRODUCT:PD-(D/E)XK nuclease superfamily protein	PI:UDG73285.1 PRODUCT:nuclease superfamily protein
PI:UCR96571.1 PRODUCT:hypothetical protein	PI:QIG59284.1 PRODUCT:hypothetical protein	PI:ATS93269.1 PRODUCT:hypothetical protein	PI:QQV88072.1 PRODUCT:hypothetical protein	PI:UDG73284.1 PRODUCT:hypothetical protein
PI:UCR96572.1 PRODUCT:13.88 kDa late protein	PI:QIG59283.1 PRODUCT:13.88 kDa late protein	PI:ATS93270.1 PRODUCT:hypothetical protein	PI:QQV88074.1 PRODUCT:hypothetical protein	PI:UDG73283.1 PRODUCT:hypothetical protein
PI:QVR48606.1 PRODUCT:transcriptional repressor	PI:QIG59282.1 PRODUCT:transcriptional repressor	PI:ATS93272.1 PRODUCT:transcriptional repressor	PI:QQV88075.1 PRODUCT:helix-trun-helix transcriptional regulator	PI:UDG73282.1 PRODUCT:hypothetical protein
PI:UCR96573.1 PRODUCT:replicative DNA helicase	PI:QIG59281.1 PRODUCT:replicative helicase/primase	PI:ATS93274.1 PRODUCT:putative replicative helicase/primease	PI:QQV88076.1 PRODUCT:putative helicase-primase	PI:UDG73281.1 PRODUCT:DNA helicase
PI:UCR96575.1 PRODUCT:helix-turn-helix domain protein	PI:QIG59278.1 PRODUCT:helix-turn-helix domain protein	PI:ATS93277.1 PRODUCT:helix-turn-helix domain protein	PI:QQV88078.1 PRODUCT:helix-turn-helix domain-containing protein	PI:UDG73279.1 PRODUCT:hypothetical protein

PI:QVR48607.1 PRODUCT:hypothetical protein	PI:QIG59277.1 PRODUCT:hypothetical protein	PI:ATS93278.1 PRODUCT:hypothetical protein	PI:QQV88079.1 PRODUCT:hypothetical protein	PI:UDG73277.1 PRODUCT:hypothetical protein
PI:QVR48610.1 PRODUCT:hypothetical protein	PI:QIG59273.1 PRODUCT:hypothetical protein	PI:ATS93281.1 PRODUCT:hypothetical protein	PI:QQV88083.1 PRODUCT:hypothetical protein	PI:UDG73274.1 PRODUCT:hypothetical protein
PI:QVR48614.1 PRODUCT:hypothetical protein	PI:QIG59269.1 PRODUCT:hypothetical protein	PI:ATS93284.1 PRODUCT:hypothetical protein	PI:QQV88086.1 PRODUCT:hypothetical protein	PI:UDG73268.1 PRODUCT:hypothetical protein
PI:QVR48618.1 PRODUCT:hypothetical protein	PI:QIG59339.1 PRODUCT:hypothetical protein	PI:ATS93289.1 PRODUCT:hypothetical protein	PI:QQV88089.1 PRODUCT:hypothetical protein	PI:UDG73265.1 PRODUCT:hypothetical protein
PI:QVR48621.1 PRODUCT:class I holin-like protein	PI:QIG59336.1 PRODUCT:class I holin-like protein	PI:ATS93291.1 PRODUCT:putative class I holin-like protein	PI:QQV88090.1 PRODUCT:class I holin-like protein	PI:UDG73262.1 PRODUCT:hypothetical protein
PI:QVR48622.1 PRODUCT:endolysin	PI:QIG59335.1 PRODUCT:putative endolysin	PI:ATS93292.1 PRODUCT:lysozyme/endolysin	PI:QQV88091.1 PRODUCT:lysozyme	PI:UDG73261.1 PRODUCT:endolysin
PI:QVR48624.1 PRODUCT:hypothetical protein	PI:QIG59334.1 PRODUCT:hypothetical protein	PI:ATS93293.1 PRODUCT:hypothetical protein	PI:QQV88093.1 PRODUCT:hypothetical protein	PI:UDG73260.1 PRODUCT:head protein
PI:QVR48626.1 PRODUCT:hypothetical protein	PI:QIG59332.1 PRODUCT:hypothetical protein	PI:ATS93295.1 PRODUCT:hypothetical protein	PI:QQV88096.1 PRODUCT:DUF2737 family protein	PI:UDG73259.1 PRODUCT:hypothetical protein
PI:QVR48627.1 PRODUCT:hypothetical protein	PI:QIG59331.1 PRODUCT:Nin protein	PI:ATS93296.1 PRODUCT:hypothetical protein	PI:QQV88097.1 PRODUCT:hypothetical protein	PI:UDG73258.1 PRODUCT:hypothetical protein
PI:QVR48628.1 PRODUCT:hypothetical protein	PI:QIG59330.1 PRODUCT: hypothetical protein	PI:ATS93297.1 PRODUCT:hypothetical protein	PI:QQV88098.1 PRODUCT:hypothetical protein	PI:UDG73257.1 PRODUCT:hypothetical protein
PI:QVR48629.1 PRODUCT:hypothetical protein	PI:QIG59329.1 PRODUCT: hypothetical protein	PI:ATS93298.1 PRODUCT:hypothetical protein	PI:QQV88099.1 PRODUCT:hypothetical protein	PI:UDG73256.1 PRODUCT:hypothetical protein
PI:QVR48631.1 PRODUCT:hypothetical protein	PI:QIG59328.1 PRODUCT: transcriptional regulator	PI:ATS93300.1 PRODUCT:hypothetical protein	PI:QQV88102.1 PRODUCT:NinH protein	PI:UDG73254.1 PRODUCT:hypothetical protein
PI:QVR48633.1 PRODUCT:terminase small subunit	PI:QIG59326.1 PRODUCT:terminase small subunit	PI:ATS93302.1 PRODUCT:putative terminase small subunit	PI:QQV88023.1 PRODUCT:small subunit of terminase	PI:UDG73251.1 PRODUCT:terminase small subunit

Table S4: Antibiotic sensitivity, the serotyping profile of 50 MDR Uropathogenic E. coli panel strains and host range testing of phage 590B using spot assay.

Sr .No.	Strain name	Cefotaxime	Cefoparazone	Gentamycin	Amikacin	Nalidixic acid	Norfloxacin	Ciprofloxacin	Nitrofurantoin	Imipenem	Cotrimoxazole	Cefoparazone+ sulbactam	Colistin	Serotyppes	Phage 590B activity
1	30608	R	R	R	R	R	R	R	S	I	R	R	S	O20	-
2	43836	R	R	R	R	R	R	R	S	R	R	R	S	O04	-
3	590	R	R	R	R	R	R	R	S	I	R	R	S	O20	++
4	40329	S	R	S	R	R	S	R	S	I	R	R	S	O83	-
5	38036	R	R	R	R	R	R	R	R	I	R	R	R	O49	-
6	15286	R	R	R	R	R	R	R	S	R	R	R	S	O135	-
7	39937	R	R	R	R	R	R	R	R	R	R	R	S	O101	-
8	1184	R	R	R	R	R	R	R	S	R	R	R	S	UT	-
9	180	R	R	R	R	R	R	R	R	R	R	R	S	UT	-
10	22895	R	R	R	S	R	R	R	R	S	R	R	S	O149	++
11	43181	R	R	R	R	R	R	R	S	R	R	R	S	O26	-
12	531	R	R	R	S	R	R	R	S	I	R	R	S	UT	-
13	AN11.3	R	R	S	R	R	R	R	S	R	R	R	S	O101	-
14	32797	R	R	R	R	R	R	R	S	R	R	R	S	O120	-
15	24331	R	R	R	R	R	R	R	I	R	R	R	S	O8	-
16	33448	R	R	R	R	R	R	R	R	I	R	R	R	UT	-

17	42383	R	R	R	R	R	R	R	S	R	S	S	S	O119	-
18	43649	S	R	S	R	R	R	R	R	S	R	R	S	UT	+
19	4183	R	R	S	S	R	R	R	S	R	R	R	S	UT	-
20	3919	R	R	R	R	R	R	R	R	R	R	R	S	O101	++
21	34449	R	R	R	R	R	R	R	R	S	S	R	S	O34	-
22	38807	R	R	R	R	R	R	R	S	S	R	R	S	O84	-
23	44649	S	R	R	R	R	R	R	R	S	R	R	S	UT	-
24	42829	R	R	R	R	R	R	R	R	R	R	R	R	O101	-
25	39854	R	R	R	R	R	R	R	R	S	S	R	S	O34	-
26	42307	R	R	R	S	R	R	R	S	S	R	R	S	O154	-
27	8824	R	S	R	S	R	R	R	S	I	R	S	S	O120	-
28	43839	R	R	R	R	R	R	R	R	R	S	S	S	O20	-
29	1195	R	R	S	S	R	R	R	S	S	R	R	S	O121	
30	22044	R	R	R	R	R	R	R	S	R	R	R	S	O34	-
31	40137	R	R	R	R	R	R	R	R	R	R	R	R	O101	-

32	38929	S	R	R	R	R	S	R	S	R	R	R	S	O11	-
33	40354	R	S	R	S	R	R	R	S	I	R	S	S	O120	-
34	44383	R	R	R	R	S	R	R	R	R	R	R	S	O11	-
35	40314	R	R	R	R	R	R	R	R	I	R	R	R	O8	-
36	191	R	R	S	S	R	R	R	S	R	R	R	S	O128	-
37	22844	R	R	S	S	R	R	R	R	S	R	R	S	O11	-
38	859	R	R	R	S	R	R	R	S	I	R	R	S	O7	-
39	40036	R	R	R	S	R	R	R	R	R	R	R	S	O149	-
40	41329	R	R	R	R	R	R	R	R	R	R	S	S	O149	-
41	41677	R	S	R	R	R	R	R	R	R	R	S	S	O14	-
42	33745	R	R	R	R	R	R	R	R	R	R	S	S	O149	-
43	39677	R	R	S	S	R	R	R	S	R	R	R	S	UT	-
44	39560	R	R	R	R	R	R	R	R	R	R	R	S	O135	-
45	40670	R	R	S	R	R	S	R	S	S	S	R	S	O135	-
46	39517	R	R	R	R	R	R	R	S	R	S	S	S	O119	-

47	42471	R	R	S	R	R	R	R	S	R	R	S	S	O83	-
48	44883	R	R	R	R	R	R	R	R	R	R	R	R	O26	-
49	41642	R	R	R	S	R	R	R	S	I	S	R	S	O120	-
50	13019	R	R	R	S	R	R	R	S	I	S	R	S	O26	-

R-resistant,I-intermediate,S-sensitive,++ clear or good lytic activity,- no activity

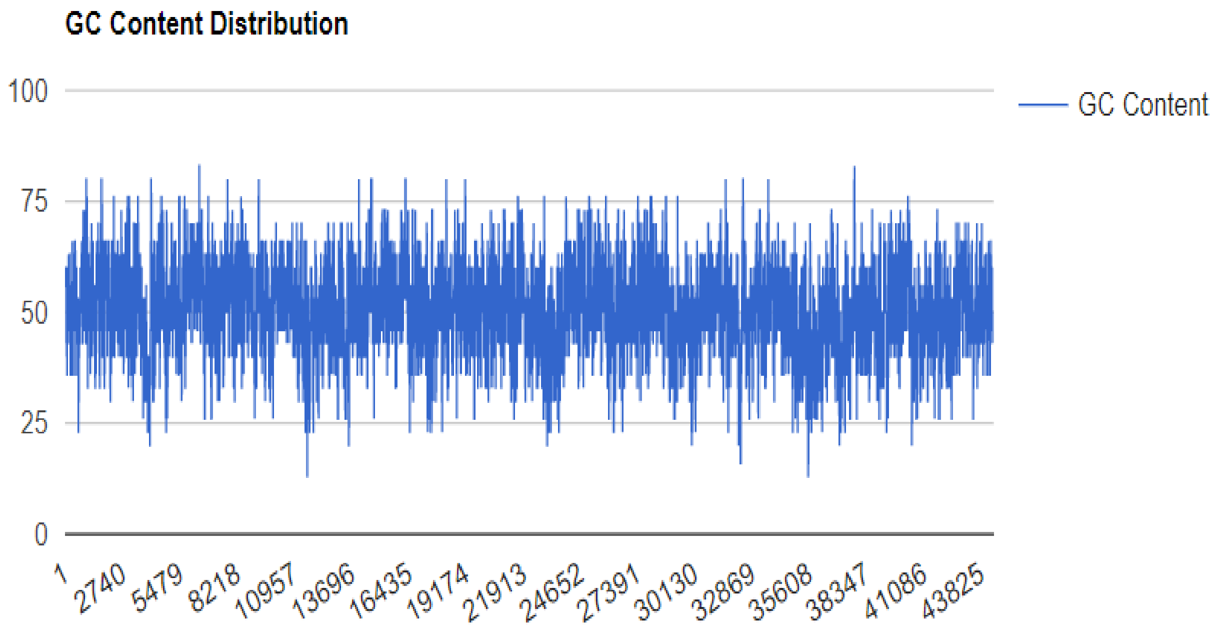

Figure S1: GC content distribution in the genome of phage 590B.

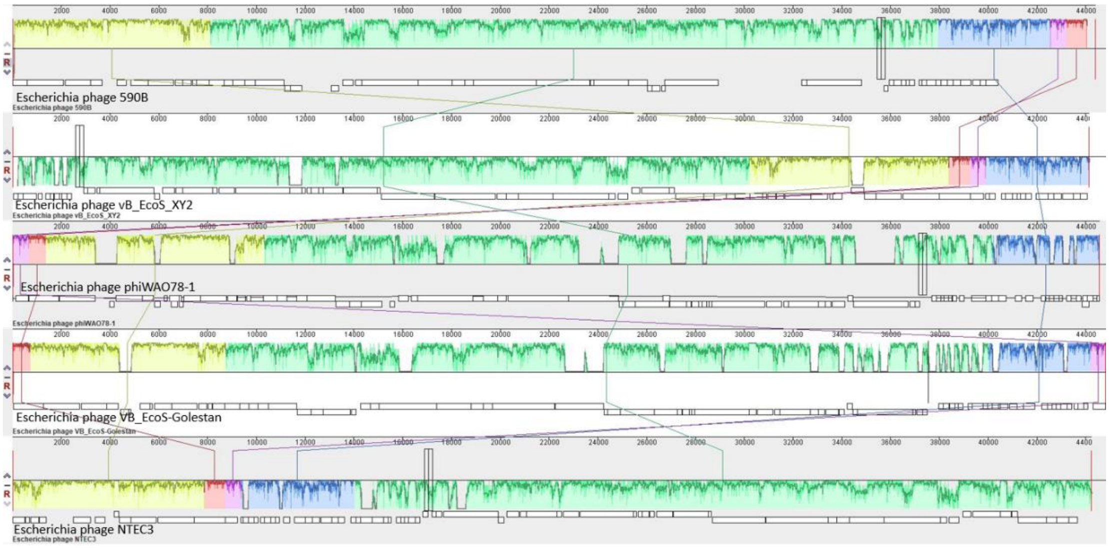

Figure S2. Genome comparison between phage *Escherichia* phage 590B and four related phages with Mauve 2.0. The degree of genome sequence similarity between the aligned region is indicated by the height of the similarity profile. The colored blocks are connected by lines depicting the homologous regions among 590B and four related phages.

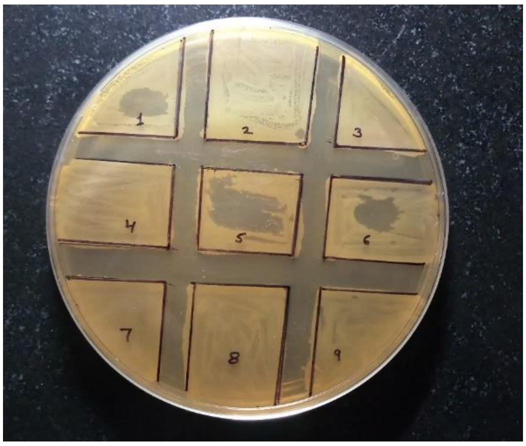

Figure S3: Representative image of spot assay used to test host range of phage 590B.

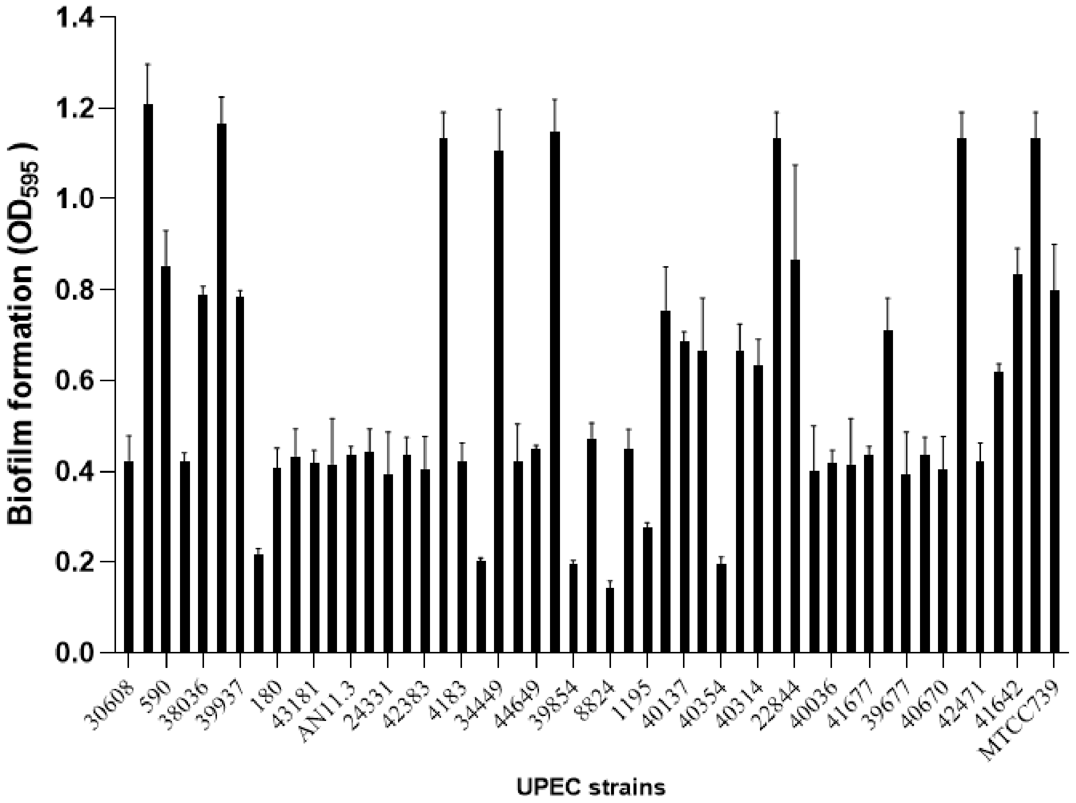

Figure S4: Biofilm formation capacity of 50 MDR, XDR UPEC strains and MTCC 739 strain in crystal violet assay.

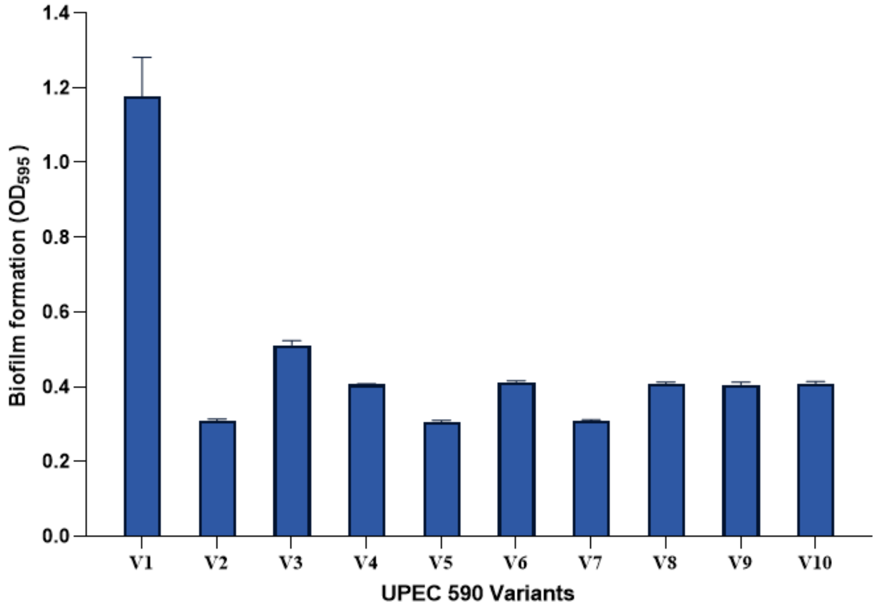

Figure S5: Biofilm-forming capacity of ten UPEC590 variants (V1-V10) in crystal violet assay.
